# Identification of Genetic Determinants of the Sexual Dimorphism in CNS Autoimmunity

**DOI:** 10.1371/journal.pone.0117993

**Published:** 2015-02-11

**Authors:** Frank Bearoff, Laure K. Case, Dimitry N. Krementsov, Emma H. Wall, Naresha Saligrama, Elizabeth P. Blankenhorn, Cory Teuscher

**Affiliations:** 1 Department of Microbiology and Immunology, Drexel University College of Medicine, Philadelphia, Pennsylvania, 19129, United States of America; 2 Department of Medicine, University of Vermont, Burlington, Vermont, 05405, United States of America; 3 Department of Pathology, University of Vermont, Burlington, Vermont, 05405, United States of America; Friedrich-Alexander University Erlangen, GERMANY

## Abstract

Multiple sclerosis (MS) is a debilitating chronic inflammatory disease of the nervous system that affects approximately 2.3 million individuals worldwide, with higher prevalence in females, and a strong genetic component. While over 200 MS susceptibility loci have been identified in GWAS, the underlying mechanisms whereby they contribute to disease susceptibility remains ill-defined. Forward genetics approaches using conventional laboratory mouse strains are useful in identifying and functionally dissecting genes controlling disease-relevant phenotypes, but are hindered by the limited genetic diversity represented in such strains. To address this, we have combined the powerful chromosome substitution (consomic) strain approach with the genetic diversity of a wild-derived inbred mouse strain. Using experimental allergic encephalomyelitis (EAE), a mouse model of MS, we evaluated genetic control of disease course among a panel of 26 consomic strains of mice inheriting chromosomes from the wild-derived PWD strain on the C57BL/6J background, which models the genetic diversity seen in human populations. Nineteen linkages on 18 chromosomes were found to harbor loci controlling EAE. Of these 19 linkages, six were male-specific, four were female-specific, and nine were non-sex-specific, consistent with a differential genetic control of disease course between males and females. An MS-GWAS candidate-driven bioinformatic analysis using orthologous genes linked to EAE course identified sex-specific and non-sex-specific gene networks underlying disease pathogenesis. An analysis of sex hormone regulation of genes within these networks identified several key molecules, prominently including the MAP kinase family, known hormone-dependent regulators of sex differences in EAE course. Importantly, our results provide the framework by which consomic mouse strains with overall genome-wide genetic diversity, approximating that seen in humans, can be used as a rapid and powerful tool for modeling the genetic architecture of MS. Moreover, our data represent the first step towards mechanistic dissection of genetic control of sexual dimorphism in CNS autoimmunity.

## Introduction

Multiple sclerosis (MS), a chronic inflammatory disease of the central nervous system (CNS) characterized by myelin loss, varying degrees of axonal damage, and progressive neurological dysfunction [1], is the most common disabling neurologic disease of young adults and adolescents, affecting 2.3 million individuals worldwide (www.atlasofms.org). MS prevalence is ~3 fold higher in females, although the disease course in men typically presents as a more rapid severe progressive disease [2]. The etiopathogenesis of MS is largely unknown; however, it is clear that it involves both genetic and environmental factors [3]. The heritability of MS is estimated to be ~30%, largely associated with the inheritance of susceptible HLA haplotypes that are present in up to 70% of MS cases [4]. However, polymorphisms in multiple other genes have also been associated with disease in genome-wide association studies (GWAS) and together contribute to 20% of the genetic variance underlying disease risk [5], suggesting that an HLA-dependent polygenic model with multiple minor interacting loci underlies the genetic component of MS susceptibility. In the GWAS analyses, the contribution of each of these genes is small (often <1%), thus both the importance of their roles in pathogenesis and the mechanisms by which different alleles act are difficult to test. Consequently, mouse models of MS are required for discerning the identities and effects of MS-GWAS gene candidates, and understanding the genetic architecture of autoimmune inflammatory disease of the central nervous system (CNS) from an evolutionary perspective.

Experimental allergic encephalomyelitis (EAE), the principal autoimmune model of MS, is elicited by sensitization of genetically susceptible animals with myelin antigens in conjunction with adjuvants [6]. In mice, the strain, immunogen, and adjuvants used determine whether the clinical signs follow an acute, chronic or remitting-relapsing disease course [7,8]. For example, immunization of SJL mice with the proteolipid protein peptide 139–151 elicits primarily a remitting-relapsing disease course, whereas immunization of C57BL/6 (B6) mice with the myelin oligodendrocyte glycoprotein (MOG) peptide 35–55 results in chronic-progressive EAE. Although CD4^+^ T cells are the primary effectors in both models, differences do exist with respect to the role of anti-myelin autoantibodies and CD8^+^ T cells in the pathogenesis of MOG-induced EAE [8]. Remarkably, given that B6 mice are the ‘genome’ strain, and are widely used for both EAE and reverse genetics, there are no known genetic studies mapping either quantitative or binary trait loci (QTL/BTL) controlling MOG-induced EAE in B6 mice.

QTL underlying natural variation require map-based cloning for their sequence identification and delineation of the causative quantitative trait nucleotides (QTNs) [9,10]. This approach is lengthy, and involves the identification of parental strains that differ in the trait of interest, establishment of a segregating population, phenotyping and genotyping of progeny, and QTL-based linkage analysis. Subsequently, physical mapping by marker-assisted selection on a pure genetic background is required to confirm the existence of a QTL and to begin the process of high resolution interative congenic mapping, where the physical location of the QTL is restricted and the number of candidate genes and potential QTNs are significantly reduced. Consomic lines, also known as chromosome (Chr) substitution strains, are unique in that they allow for accelerated detection of QTL/BTLs by direct genome-wide physical mapping [11]. Moreover, the existence of consomic strains with different wild-derived diploid Chrs and conplastic strains with mitochondrial DNA on a pure genetic background, such as B6, overcomes the relative lack of genetic diversity inherent in classical laboratory inbred strains [12], and more accurately reflects the greater evolutionarily selected genetic diversity seen in humans.

PWD/PhJ mice are an inbred strain descended from a pair of *M*. *m*. *musculus* wild mice trapped in 1974. It is estimated that *M*. *m*. *musculus* and *M*. *m*. *domesticus*, the subspecies to which classical laboratory strains including B6 belong, diverged roughly 350,000 years ago[13]. These subspecies capture 70–80% of known variation that is almost as great as *M*. *spretus* (84%), which diverged approximately 3 million years ago [14,15]. The genetic diversity between B6 and PWD mice is ~55% compared to ~12% among classical laboratory inbred strains (http://phenome.jax.org/). Moreover, compared to classical laboratory inbred strains, wild-derived strains are, in general, more resistant to a variety of pathogens, most notably viral infections [16,17]. In comparison to B6, PWD mice have proportionally more neutrophils and total CD4^+^ and CD8^+^ T cells but decreased B cells and NK cells (http://phenome.jax.org/ and [18]). Consomic B6 mice bearing single Chrs from the PWD strain typically display a continuous distribution of these phenotypes [18]. Lastly, these two strain share the same H2^b^ haplotype allowing analysis of genetic variation on disease independent of effects from MHC differences (http://jaxmice.jax.org/literature/catalog/mhc_h2_haplotypes.pdf).

In this report, we studied a panel of 26 C57BL/6J-Chr#^PWD/PhJ^/ForeJ (B6-Chr#^PWD/PhJ^) consomic and sub-consomic strains of mice to identify QTL/BTL controlling MOG-induced EAE in B6 mice. Nineteen linkages on 18 chromosomes exhibited evidence of harboring QTL/BTL controlling MOG-EAE. Of these 19 linkages, six were male-specific, four were female-specific, and nine were non-sex-specific, consistent with a differential genetic control of disease course between males and females. An MS-GWAS candidate-driven bioinformatic analysis using orthologous genes linked to EAE susceptibility identified sex-specific and non-sex-specific gene networks underlying disease pathogenesis. An analysis of sex hormone regulation of genes within these networks identified several key molecules, prominently including the MAP kinase family, known hormone-dependent regulators of sex differences in EAE susceptibility [19,20]. Importantly, our results provide the framework by which consomic mouse strains with overall genome-wide genetic diversity, approximating that seen in humans, can be used as a rapid and powerful tool for modeling the genetic architecture of MS. Moreover, our data represent the first step towards mechanistic dissection of genetic control of sexual dimorphism in CNS autoimmunity.

## Results and Discussion

### MOG-EAE in B6-Chr#^PWD/PhJ^ consomic mice is sexually dimorphic

Sexual dimorphisms in susceptibility to EAE and clinical disease course are well documented; however, conflicting results have been seen with MOG-induced EAE [21–23], and to our knowledge, a detailed study examining the EAE sexual dimorphism in B6 mice elicited using the 2× MOG_35–55_+CFA protocol (that does not include pertussis toxin as an ancillary adjuvant) has not been reported. Therefore, we performed an analysis of MOG-EAE in 8–12 wk old female and male B6 mice elicited using the 2× MOG_35–55_+CFA protocol [24]. Overall, compared to B6 female mice, B6 male mice were significantly more susceptible to MOG-EAE elicited by 2× MOG_35–55_+CFA immunization (**S1 Fig.**).

**Fig 1:**
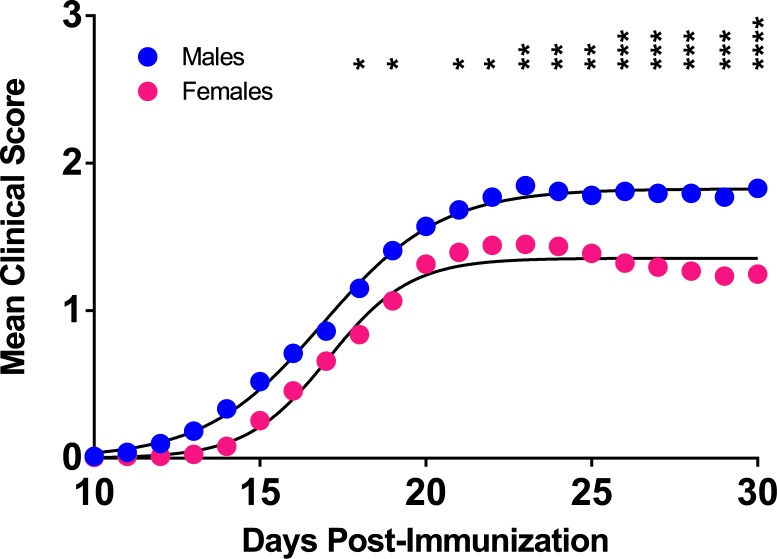
Clinical disease course of MOG-EAE in consomic strains is sexually dimorphic. A composite female and male clinical disease course for all consomic strains (N≥149 per sex, N≥5 per strain) studied was generated using the mean daily clinical scores. The significance of the observed differences in disease course severity was determined by two-way ANOVA followed by Bonferroni multiple comparison test. A significant effect of sex (p<0.0001), days post-immunization (p<0.0001), and sex-by-days post-immunization interaction term (p<0.0001) were detected with * p<0.05; ** p<0.01; *** p<0.001; **** p<0.0001.

**Fig 2:**
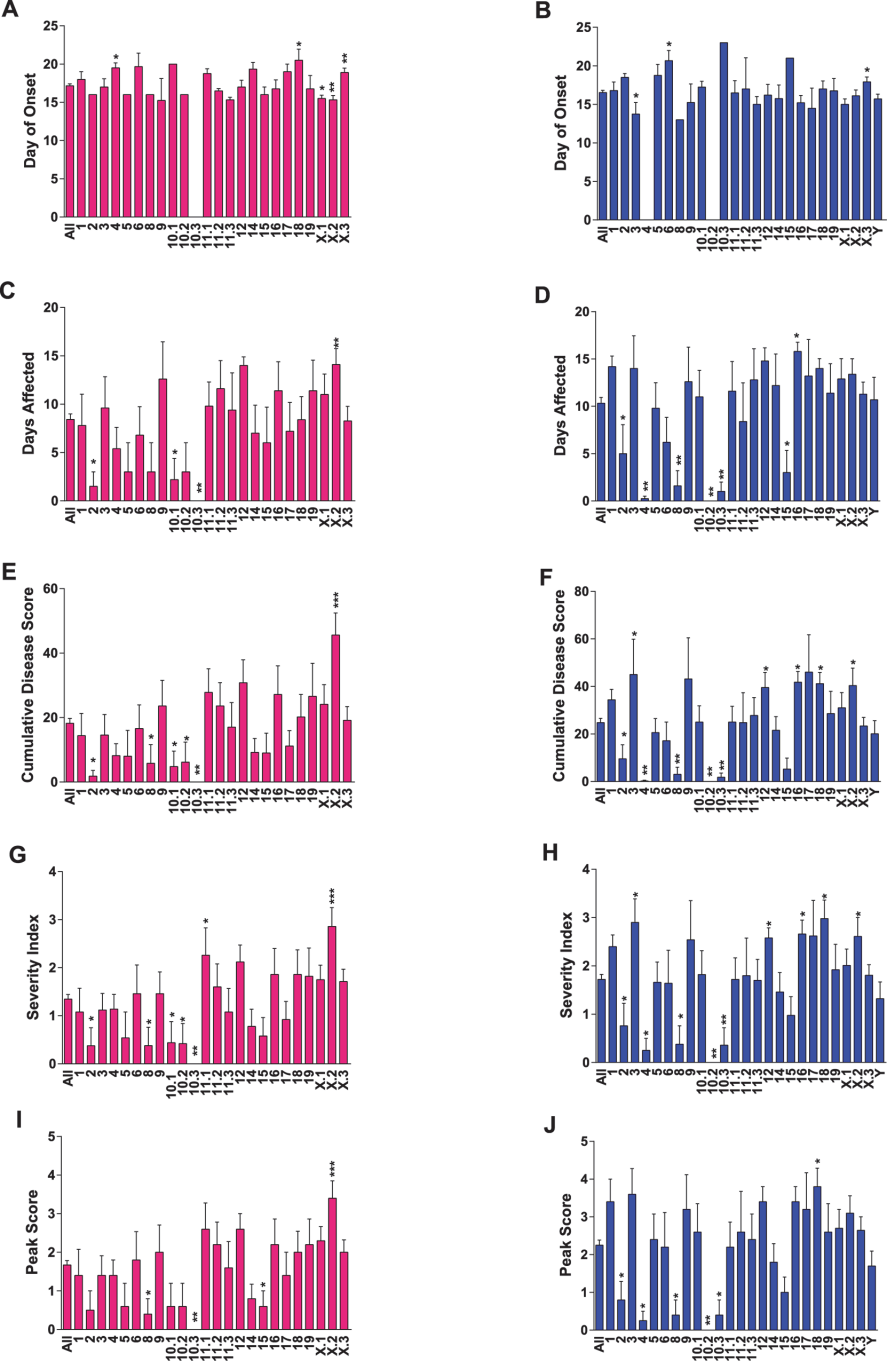
Clinical quantitative traits controlling MOG-EAE in female and male B6-Chr#^PWD/PhJ^ strains. (A-B) day of onset (DO), (C-D) days affected (DA), (E-F) cumulative disease score (CDS), (G-H) severity index (SI), and (I-J) peak score (PS). Females in left column (pink) and males in right column (blue). The significance of the observed differences in clinical trait variables among female and male strains was determined using the Mann-Whitney U test of each strain against the mean trait variable for all strains by sex. *, p<0.05; * p<0.01; ***, p<0.001; ****, p<0.0001.

**Fig 3:**
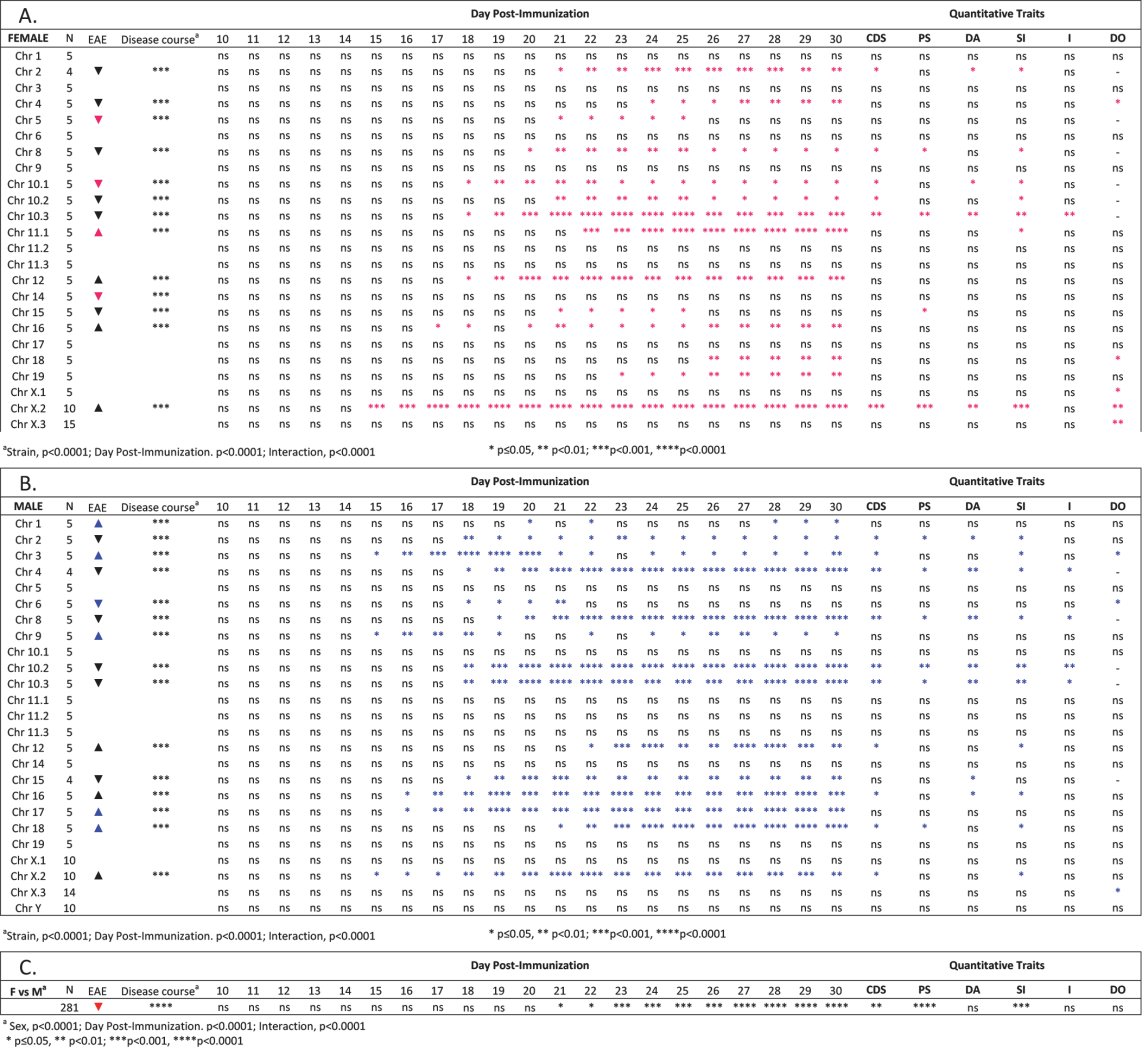
Physical mapping of QTL controlling MOG-EAE in female and male B6-Chr#^PWD/PhJ^ strains of mice. Cohorts of (**A**) female and (**B**) male mice were immunized for the induction of EAE using the 2×MOG_35–55_+CFA protocol. The significance of the observed differences in disease course severity among the strains was determined by two-way ANOVA with post hoc multiple comparisons tests of female and male strains against the composite disease course (**C**) for all female and male strains respectively. The significance of observed differences in disease course, cumulative disease score (CDS), days affected (DA), severity index (SI), peak score (PS), incidence (I), and day of onset (DO) was determined using the Mann-Whitney U test of female and male strain against the corresponding quantitative trait variables for all female and male strains studied, respectively. Arrows indicate the direction of the change in disease course severity; red symbols for female-specific QTL, blue symbols for male-specific QTL, and black symbols for non-sex-specific QTL are shown.

We next studied the susceptibility of B6-Chr#^PWD/PhJ^ consomic and sub-consomic strains to MOG-EAE. Given the EAE sexual dimorphism in B6 mice (**S1 Fig.**), the EAE data for female and males were analyzed separately. Like B6 mice, disease course for the entire B6-Chr#^PWD/PhJ^ consomic panel was sexually dimorphic, with greater disease seen in males (Fig. 1). Clinical quantitative trait variables for each strain largely followed disease course and are detailed in Fig. 2 and summarized in Fig. 3. When a significant effect on overall disease course of a Chr or Chr-interval was detected in one sex but not the other, it was designated as a sex-specific-QTL/BTL controlling disease course. Female-specific QTL/BTL mapped to Chrs 5, 10.1, 11.1, and 14 (Fig. 3A), while Chrs 1, 3, 6, 9, 17, and 18 were male-specific (Fig. 3B). When a significant effect on overall disease course was detected in both males and females, it was designated as non-sex-specific, and mapped to Chrs 2, 4, 8, 10.2, 10.3, 12, 15, 16, and X.2. Little or no evidence was detected in support of EAE-QTL/BTL on Chr 19 and Y or on Chr intervals 11.2, 11.3, X.1, and X.3.

Of the female-specific EAE-QTL/BTL, all except for Chr 11.1 were associated with decreased disease severity. In contrast, all the male-specific linkages were associated with increased disease severity, with the exception of Chr 6. Non-sex-specific EAE-QTL/BTL residing on Chrs 12, 16, and X.2 were associated with increased disease severity while Chrs 2, 4, 8, 10.2, 10.3, 15 decreased severity. Thus, a total of 9/19 linkages showed increased disease course associated with PWD alleles. More striking is the finding that the genetic load underlying MOG-EAE in female and male B6 mice is sexually dimorphic, with the female genetic load being less than that of males (4 female-specific linkages vs. 6 male-specific linkages). Moreover, the distribution of alleles contributing to increased disease severity is sexually dimorphic in that 1/4 female-specific QTL/BTL have PWD alleles, whereas 5/6 male-specific linkages contributing to increased disease are from PWD.

Importantly, with respect to modeling the genetic architecture of MS, we compared the genetic diversity between the B6 and PWD alleles of the orthologues of the current best list of non-MHC MS susceptibility variants [25]. It should be noted that this list of MS-GWAS candidates is not final since linkage disequilibrium has hindered identification of the particular variant and gene responsible in many regions of interest. The analysis revealed a remarkable degree of genetic diversity among the orthologous alleles (**S1 Table)**. Moreover, an analysis of the functional classification and location of the most significant MS-GWAS associated SNPs (intronic, intergenic, exonic, 3-UTR, 5-UTR, regulatory, etc.) revealed that all were similarly represented among the murine orthologues (**S2 Table**).

### MOG-EAE in B6-Chr#^PWD/PhJ^ consomic mice allows rapid and efficient genetic mapping of disease traits

The advantages of using B6-Chr#^PWD/PhJ^ consomic mice in studies for EAE QTL/BTL identification are multiple. The two parent strains are highly dissimilar, and thus, a significant portion of the genome is polymorphic and can be interrogated for the presence of EAE-modifying loci. Second, QTL or BTL congenic strains are usually constructed after expensive linkage mapping in backcrosses or F2 intercrosses; but too often, the effect of any single QTL interval is too small for positional cloning to be successful without requiring very large cohorts of congenic and control mice with the susceptible or resistant haplotypes at the QTL. In addition, necessary epistatic interactions with other donor-derived genes are unpredictable and their absence after selecting mono-interval congenic lines generally reduces the action of the selected QTL. In our previous genetic analysis of EAE QTL/BTL in congenic mice, many of these difficulties were encountered [26]. However, by testing consomic strains, one may choose mice that have a uniform B6 background, have the desired EAE phenotype, and nevertheless bear only a single Chr or portion of a Chr from the donor strain, in this case, PWD. This completely reduces all but syntenic sources of epistasis from confounding EAE QTL/BTL identification when such consomic intervals have been reduced by selective breeding.

The most useful consomics are those that carry a robust EAE QTL/BTL on the smallest PWD-derived interval. Such strains include B6-Chr10.2^PWD/PhJ^, B6-Chr10.3^PWD/PhJ^, B6-Chr11.1^PWD/PhJ^, and ChrX.2^PWD/PhJ^ mice [12]. For example, B6-Chr10.2^PWD/PhJ^ harbors a PWD-derived interval of 69.4 Mb, and B6-Chr10.3^PWD/PhJ^, an interval of 50.1 Mb. It is not yet known if the QTL/BTL detected in this study is in the shared 12.8 Mb portion of Chr 10 found in both sub-consomics, or if there are two distinct QTL/BTL on Chr 10. Contained within these two intervals are numerous genes that are likely to be highly polymorphic, including the important GWAS gene candidates, such as *Tsfm*, *Themis*, and *Ptprk* among others [25], or are crucial players in inflammatory processes, such as *Ifng*, *Il22*, *Stat2*, and *Stat6*. These genes can be separated by recombination in subsequent generations to delineate their role in processes known to be important to MS and EAE pathogenesis.

### *In Silico* Analysis Reveals the Influence of Sex Hormone-Dependent Signaling Pathways on Genetic Control of the EAE Sexual Dimorphism

**Fig 4:**
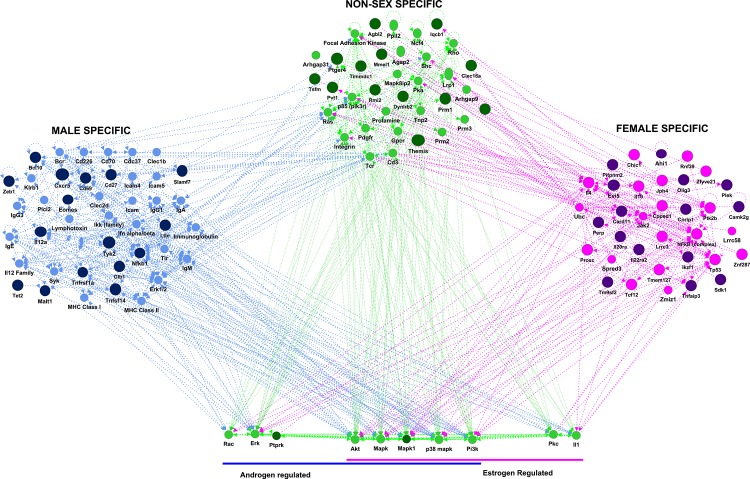
Influence of sex hormone-dependent signaling pathways on genetic control of the EAE sexual dimorphism. Gene lists were generated using the polymorphic MS-GWAS orthologues residing within EAE-QTL/BTL that were either male-specific, female-specific, or non-sex-specific. These lists were used to identify the top associated functional gene networks using the core analysis feature in IPA (**Table 1**). GWAS candidates are dark shaded. Within these networks, we identified genes that were predicted to be regulated by estrogens and/or androgens using the connect tool in IPA’s Pathway Designer. These genes included *Rac*, *Erk*, *Pkc*, *Il-1*, *Akt*, *Pi3k*, and members of the MAPK family, including p38 MAPK. These genes were pulled out from the main non-sex-specific module and displayed at the bottom of the figure to more easily observe their connections to the male- and female-specific modules.

Sex hormones contribute to the sexual dimorphism in MS and EAE [27]. To tease apart the signaling pathways whereby androgens and estrogens contribute to the sexual dimorphism in our model, we utilized a network based bioinformatic approach. First, we generated gene lists of the polymorphic MS-GWAS orthologues residing within EAE-QTL/BTL that were male, female, or non-sex-specific. These lists were used to identify the top associated functional gene networks (**Table 1 and Fig. 4**). Next, within these networks, we identified genes that were predicted to be regulated by estrogens and/or androgens. These genes included *Rac*, *Erk*, *Pkc*, *Il-1*, *Akt*, *Pi3k*, and members of the MAPK family, including p38 MAPK. The MAPK family is the largest group of interactors, and this is especially striking for p38 MAPK, a kinase shown by us and others to be central to many aspects of EAE pathogenesis [20]. Most strikingly, our recent results indicate that p38 MAPK signaling can control EAE pathogenesis in a hormone-dependent, sexually dimorphic fashion [19]. Importantly, the MAPK family interacts extensively with the male- and female-specific gene networks (**Fig. 4**). Taken together, these findings support our model whereby p38 and other MAPKs function as a central node integrating hormonal signals to regulate the genetic control of the sexual dimorphism in CNS autoimmunity.

**Table 1:** Top signaling networks in male-specific, non-sex-specific, and female-specific gene lists in IPA and constituent molecules.

Gene Set	Score^a^	Top Diseases and Functions	Focus Molecules^b^	Molecules in Network^c^
MSS	37	Cellular Development, Hematological System Development and Function, Hematopoiesis	16	**BCL10**, BCR (complex), **CD27**, **CD69**, CD70, CD226, CDC37, CLEC1B, CLEC2D, **CXCR5**, **EOMES**, ERK1/2, **GLB1**, ICAM4, ICAM5, Icam, IFN alpha/beta, Iga, Ige, IgG1, Igg3, Igm, Ikk (family), IL12 (complex), IL12 (family), **IL12A**, Immunoglobulin, KLRB1, **LTBR**, Lymphotoxin, **MALT1**, MHC Class I (complex), MHC Class II (complex), **NFKB1**, PLCL2, **SLAMF7**, SYK/ZAP, **TET2**, Tlr, **TNFRSF1A**, **TNFSF14**, **TYK2**, **ZEB1**
	32	Cellular Growth and Proliferation, Nervous System Development and Function, Cell Morphology	15	APP, ARMC9, C18orf21, C1orf106, C9orf41, C9orf142, CEBPA, CHAC2, CHID1, **CMC1**, **CTSH**, **DDAH1**, EPO, **EXTL2**, **FNDC1**, **FOXP1**, G protein beta gamma, HINT3, Histone h4, HTT, **JAZF1**, **MORF4L1**, NFkB (complex), **PHGDH**, PUS7L, RABL3, RNASE11, SFN, **SLC44A2**, **SOX8**, **SP140**, TBCC, UBC, **ZC2HC1A**, **ZFP36L2**
	20	Hematological System Development and Function, Tissue Morphology, Cellular Development	10	Akt, Ap1, caspase, **CCR4**, CD3, **DDX6**, **EOMES**, ERK, Gpcr, Histone h3, HLA-DRB1, IKK (complex), IL23, IL12 (family), Interferon alpha, Jnk, Mapk, **NDFIP1**, P38 MAPK, Pdgf (complex), PDGF BB, PI3K (complex), Pkc(s), Pro-inflammatory Cytokine, Ras, **RGS1**, **SATB1**, **SKAP2**, **STAT4**, STAT5a/b, TCR, Tlr, Tnf (family), **VCAM1**, Vegf
	11	Molecular Transport, Nucleic Acid Metabolism, Small Molecule Biochemistry	6	AGPAT1, CELSR1, D-glucose, GATA4, GBP5, GPR12, GPR19, GPR64, GPR85, GPR108, GPR123, GPR146, GPR156, GPR158, GPRC5B, **HAAO**, **HOXA1**, HRH4, IFNG, LPAR4, LZTS1, **MANBA**, Mbl1, mir-375, NPBWR1, P2RY14, Pka, **PKIA**, SLC28A2, SLC52A2, SLC5A2, **SSTR5**, TACR2, **TREH**, trehalose
NSS	32	Cellular Development, Cellular Growth and Proliferation, Cellular Movement	15	AGAP2, **AGBL2**, AKT, ARHGAP9, ARHGAP31, **CAPSL**, CD3, **CLEC16A**, **DYNLRB2**, ERK, Focal adhesion kinase, Gpcr, IL1, Integrin, **IQCB1**, LRP1, MAPK8IP2, **MMEL1**, NCF4, p38 MAPK, **MAPK1**, p85 (pik3r), Pdgfr, PI3K, Pka, Pkc, PPIL2, **PRM1**, PRM2, PRM3, Protamine, **PTGER4**, **PTPRK**, PVT1, RAC, Ras, Ras homolog, RMI2, Shc, TCR, **THEMIS**, **TIMMDC1**, TNP2, TSFM
	14	Cell-To-Cell Signaling and Interaction, Cell Death and Survival, Cellular Compromise	7	**AGBL2**, APP, AR, ARMC9, C18orf21, C9orf142, **DYNLRB2**, HINT3, **IQCB1**, JAK2, MROH1, **PLEKHG5**, PUS7L, **PVT1**, RHOA, RMI2, RNASE11, RNF39, SVOPL, TBCC, TP53, **TSFM**, UBC
	12	Cellular Function and Maintenance, Hematological System Development and Function, Tissue Morphology	6	**BACH2**, **CYP24A1**, **DAB2**, Fc gamma receptor, Gm-csf, HLA-DR, Ifn, IFN alpha/beta, IFN Beta, Ifn gamma, Ifnar, Iga, IgG1, Igg3, IgG2a, IgG2b, IL12 (complex), Immunoglobulin, IRF, **IRF8**, JAK, MHC Class II (complex), **SOCS1**, TCF, Tlr, **TYMP**, **ZBTB46**
FSS	48	Cellular Growth and Proliferation, Cancer, Hematological Disease	16	**AHI1**, **CAMK2G**, **CARD11**, CHIC1, CNRIP1, CPPED1, **EVI5**, **IKZF1**, IL4, IL19, **IL20RA**, **IL22RA2**, JAK2, JPH4, LRRC3, NFkB (complex), **OLIG3**, **PERP**, **PITPNM2**, PLEK, PROSC, PTK2B, RNF39, **SDK1**, SPRED3, TCF12, **TM9SF2**, TMEM127, **TNFAIP3**, TP53, UBC, ZFYVE21, **ZMIZ1**, ZNF287

^a^ IPA generated p-score from molecules in network; higher scores indicate more significance

^b^ molecules from gene list in network

^c^ molecules originating from the MS-GWAS list are bolded

### Concluding Remarks

Despite the well-documented sexual dimorphism in MS incidence and progression, little is known about the underlying genetic mechanisms. We have employed MOG-induced EAE, a mouse model of MS, in consomic B6-Chr#^PWD/PhJ^ mice to map disease-specific QTL/BTL, and to evaluate the sex differences inherent to each. Use of consomic strains between B6 and the wild-derived inbred PWD strain allowed us to overcome the lack of genetic diversity in classical inbred laboratory strains to better reflect the greater evolutionarily selected genetic diversity of humans, to model MS genetics. The vast majority of the orthologous MS-GWAS candidates are polymorphic between B6 and PWD mice (**S2 Table**). We find (1) sexual dimorphism in the consomic mice, where males are more susceptible to MOG-EAE (**Fig. 1, Fig. 3C**) and (2) utilization of the MAPK family, as a central regulatory hub for hormonal control of the sexual dimorphism in B6 mice (**Fig. 4**). Importantly, our results provide a framework in which the increased genetic diversity of wild-derived inbred consomic strains can provide a better understanding of polygenic human diseases. Such studies may identify critical pathways and networks that can be targeted for sex-specific therapeutic intervention and/or prevention in MS. Our studies suggest that readily druggable kinase and hormonal signaling pathways are plausible targets for such therapeutic intervention.

## Materials and Methods

### Mice

Male and female C57BL/6J (B6), C57BL/6J-Chr 1^PWD/Ph^/ForeJ, C57BL/6J-Chr 2^PWD/Ph^/ForeJ, C57BL/6J-Chr 3^PWD/Ph^/ForeJ, C57BL/6J-Chr 4^PWD/Ph^/ForeJ, C57BL/6J-Chr 5^PWD/Ph^/ForeJ, C57BL/6J-Chr 6^PWD/Ph^/ForeJ, C57BL/6J-Chr 8^PWD/Ph^/ForeJ, C57BL/6J-Chr 9^PWD/Ph^/ForeJ, C57BL/6J-Chr 10.1^PWD/Ph^/ForeJ, C57BL/6J-Chr 10.2^PWD/Ph^/ForeJ, C57BL/6J-Chr 10.3^PWD/Ph^/ForeJ, C57BL/6J-Chr 11.1^PWD/Ph^/ForeJ, C57BL/6J-Chr 11.2^PWD/Ph^/ForeJ, C57BL/6J-Chr 11.3^PWD/Ph^/ForeJ, C57BL/6J-Chr 12^PWD/Ph^/ForeJ, C57BL/6J-Chr 14^PWD/Ph^/ForeJ, C57BL/6J-Chr 15^PWD/Ph^/ForeJ, C57BL/6J-Chr 16^PWD/Ph^/ForeJ, C57BL/6J-Chr 17^PWD/Ph^/ForeJ, C57BL/6J-Chr 18^PWD/Ph^/ForeJ, C57BL/6J-Chr 19^PWD/Ph^/ForeJ, and C57BL/6J-Chr Y^PWD/Ph^/ForeJ (abbreviated throughout the text as either B6-Chr, Chr#, or simple #) were purchased from The Jackson Laboratory (Bar Harbor, ME) and allowed 2 weeks to acclimate to the UVM animal facility before any experimental procedures were carried out. All B6-Chr mice carry full length PWD chromosomes with the exceptions of the subconsomic lines B6-Chr 10.1, 10.2, 10.3, 11.1, 11.2, 11.3, X.1, X.2, and X.3 which carry overlapping intervals for chromosomes 10, 11, and X respectively [12]. All mice were housed at 25°C with 12:12h light-dark cycles and 40–60% humidity on Sani-Chips bedding (P.J. Murphy, Montville, NJ). The experimental procedures performed in this study were approved by the Animal Care and Use Committee of the University of Vermont.

### Induction and Evaluation of EAE

EAE was induced in healthy 8–12 week old mice as described previously [24]. Mice were injected s.c. with an emulsion containing 100 μg of MOG_35–55_ peptide (MEVGWYRSPFSRVVHLYRNGK) (Peptide core facility, University of Vermont, Burlington, VT) and complete Freund’s adjuvant (CFA) (Sigma-Aldrich, St. Louis, MO) supplemented with 200 μg of *Mycobacterium tuberculosis* H37RA (Difco Laboratories, Detroit, MI) in the posterior right and left flank; one week later all mice were similarly injected at two sites on the right and left flank anterior of the initial injection sites (2× MOG_35–55_+CFA). Injections were carried out in the home cages of all mice housed five per cage.

Mice were evaluated daily for clinical signs beginning at day 10 through day 30 as follows: 0, no clinical expression of disease; 1, flaccid tail without hind limb weakness; 2, hind limb weakness; 3, complete hind limb paralysis and floppy tail; 4, hind leg paralysis accompanied by a floppy tail and urinary or fecal incontinence; 5, hind limb paralysis and fore limb weakness, with or without incontinence, preventing the movement of the animal around the cage [24]. Mice that reached a score of 5 prior to day 30 were humanely euthanized by CO_2_ asphyxiation to prevent suffering. Napa Nectar and food pellets were placed on the floor of any cage housing mice reaching a score of 2 or greater to ensure the mice can reach food and do not become dehydrated. Quantitative traits were calculated as follows for the 20 day scoring period: cumulative disease score (CDS) is the summation of daily scores, peak score (PS) is the highest score attained, days affected (DA) is the total days in which a mouse was at a score of ≥1, day of onset (DO) was the first day at which an animal presents with a score of ≥1. Mice were considered positive for incidence (I) if they showed any clinical signs ≥1 for two or more days [28–30]. Mice were immunized in four separate cohorts, and the same lots of MOG_35–55_, complete Freund’s adjuvant, and *M*. *tuberculosis* H37Ra were used through this study.

### Gene Lists

The MS-GWAS list was composed from orthologous candidates positioned within EAE QTL/BTL [25]. These candidates were assigned to either female-specific, male-specific, or non-sex-specific gene lists based on their location within the respective EAE-QTL. These lists were then run through the batch query tool of the Mouse Genome Database (MGI, http://www.informatics.jax.org/batch) to determine genomic location and filter out invalid gene symbols. Single nucleotide polymorphisms (SNPs) between B6 and PWD within candidate genes were determined through the Mouse Phenome Database (http://phenome.jax.org/) using the CGD-MDA1, Perlegen2, and Broad2 datasets.

### Network analysis

To obtain bioinformatic insight into the gene networks influencing the sexual dimorphism of MOG-EAE in B6-PWD consomics, we ran analyses on male-specific, female-specific, and non-sex-specific lists of genes generated from **S2 Table** using the core analysis tool of Ingenuity Pathway Analysis Software (IPA) (Ingenuity Systems, http://www.ingenuity.com). The Ingenuity Knowledge Base was used as a reference, with default settings. The genes and their interactors residing in the top networks were imported into IPA’s pathway designer tool, thereby producing the male-specific, female-specific, and non-sex-specific modules. The interactions between the genes within the non-sex-specific and the male or female modules were identified using the connect tool. We then again used the connect tool to identify those genes that have known associations with androgens and/or estrogens.

### Statistical analyses of QTL/BTL for EAE

Statistical analyses were performed using GraphPad Prism version 6 (GraphPad Software, San Diego, CA, USA). The specific tests used are detailed in the figure legends and table footnotes.

## Supporting Information

S1 Fig MOG-EAE in B6 mice elicited by 2× MOG_35–55_+CFA immunization is sexually dimorphic. Disease course for male and female B6 mice (N≥21) from each of the consomic immunization cohorts. Disease course [effect of day post-immunization, extra-sum-of-squares F test (F = 45.6, DFn = 25, DFd = 1274, p<0.0001); effect of strain (F = 171.4, DFn = 1, DFd = 1274, p<0.0001); interaction (F = 6.6, DFn = 25, DFd = 1274, p<0.0001); Bonferroni multiple test comparisons: ** ≤0.01, **** ≤0.0001], incidence (I, Fisher’s exact text), days affected (DA), cumulative disease score (CDS), severity index (SI), and peak score (PS, Mann Whitney U test).
(TIF)

S1 Table Current best list of non-MHC multiple sclerosis susceptibility variants and murine orthologues with SNPs [25]. (XLSX)

S2 Table List of SNPs within MS-GWAS candidate gene murine orthologues between C57BL/6J and PWD/PhJ mouse strains. (XLSX)
