# Supplementary figures and images for: Identification of Genetic Determinants of the Sexual Dimorphism in CNS Autoimmunity
Source: PLoS One. 2015 Feb 11;10(2):e0117993. doi: 10.1371/journal.pone.0117993 (PMC4324900; doi:10.1371/journal.pone.0117993)

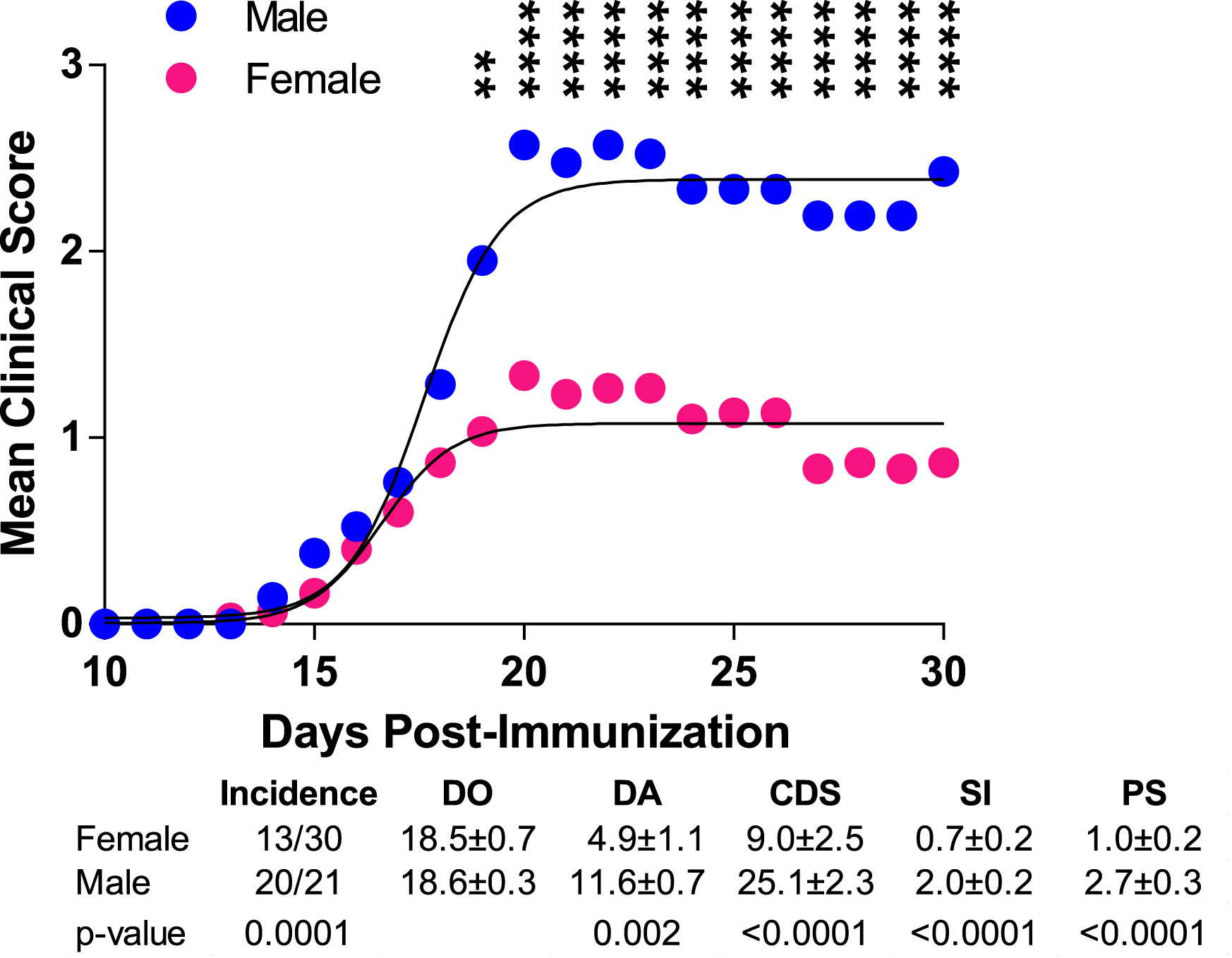

Supplement: S1 Fig — Disease course for male and female B6 mice (N≥21) from each of the consomic immunization cohorts. Disease course [effect of day post-immunization, extra-sum-of-squares F test (F = 45.6, DFn = 25, DFd = 1274, p<0.0001); effect of strain (F = 171.4, DFn = 1, DFd = 1274, p<0.0001); interaction (F = 6.6, DFn = 25, DFd = 1274, p<0.0001); Bonferroni multiple test comparisons: ** ≤0.01, **** ≤0.0001], incidence (I, Fisher’s exact text), days affected (DA), cumulative disease score (CDS), severity index (SI), and peak score (PS, Mann Whitney U test). (TIF) [file pone.0117993.s001.tif]
